# The Impact of Quality of Life on Cardiac Arrhythmias: A Clinical, Demographic, and AI-Assisted Statistical Investigation

**DOI:** 10.3390/diagnostics15070856

**Published:** 2025-03-27

**Authors:** Luiza Camelia Nechita, Ancuta Elena Tupu, Aurel Nechita, Daniel Voipan, Andreea Elena Voipan, Dana Tutunaru, Carmina Liana Musat

**Affiliations:** 1 Faculty of Medicine and Pharmacy, ‘Dunarea de Jos’ University of Galati, 800008 Galati, Romania; 2 Faculty of Automation, Computers, Electrical Engineering and Electronics, ‘Dunarea de Jos’ University of Galati, 800008 Galati, Romania

**Keywords:** quality of life, cardiac arrhythmias, depression, artificial intelligence, statistical methods, PHQ-9, SF-36

## Abstract

**Background/Objectives**: Cardiac arrhythmias impact quality of life (QoL) and are often linked to psychological distress. This study examines the relationship between QoL, depression, and arrhythmias using AI-assisted analysis to enhance patient management. **Methods**: A total of 145 patients with arrhythmias were assessed using an SF-36 health survey (QoL) and a PHQ-9 questionnaire (depression). Statistical analyses included regression, clustering, and AI-based models such as K-means and logistic regression to identify risk factors and patient subgroups. **Results**: Patients with comorbidities had lower QoL and higher depression scores. PHQ-9 scores negatively correlated with SF-36 mental health components. AI-assisted clustering identified distinct patient subgroups, with older individuals and those with longer disease duration exhibiting the lowest QoL. Logistic regression predicted depression with 93% accuracy, and XGBoost achieved an AUC of 0.97. **Conclusions**: QoL plays a key role in arrhythmia management, with depression significantly influencing outcomes. AI-driven predictive models offer personalized interventions, improving early detection and treatment. Future research should integrate wearable technology and AI-based monitoring to optimize patient care.

## 1. Introduction

### 1.1. General Context of Cardiac Arrhythmias and Quality of Life

Cardiac arrhythmias, characterized by irregular heartbeats, are among the most prevalent cardiovascular disorders worldwide [1]. These disturbances in heart rhythm, including atrial fibrillation, bradycardia, tachycardia, and extrasystoles, can range from benign to life-threatening conditions [2]. Arrhythmias affect millions of individuals globally, contributing significantly to morbidity, mortality, and healthcare costs [3].

The impact of cardiac arrhythmias extends beyond physical symptoms such as palpitations, dizziness, fatigue, and syncope. Patients often experience psychological distress, including anxiety and depression, due to the unpredictable nature of their condition and the potential for severe complications such as stroke or sudden cardiac arrest [4,5]. The chronic nature of arrhythmias and the lifelong management they require can significantly diminish patients’ quality of life (QoL) [6].

QoL has become a critical outcome measure in the management of chronic diseases, including cardiac arrhythmias. QoL encompasses physical, psychological, and social well-being, reflecting how patients perceive and cope with their condition [7,8]. In arrhythmia patients, reduced QoL is associated with symptom burden, limitations in daily activities, and emotional distress [9]. Assessing QoL is essential for comprehensive patient care, allowing healthcare providers to tailor interventions that address both the physiological and psychosocial aspects of the disorder [10].

Advances in diagnostic tools, therapeutic options, and patient-centered care models emphasize the importance of QoL assessments in clinical practice. Instruments like the Short Form-36 (SF-36) health survey [11] and Patient Health Questionnaire-9 (PHQ-9) [12] enable clinicians to measure and monitor patients’ health status and mental well-being, facilitating personalized treatment plans aimed at improving overall QoL. Understanding the interplay between cardiac arrhythmias and QoL is vital for enhancing patient outcomes and optimizing long-term care strategies [13].

### 1.2. Impact of Depression and Mental Health on Cardiovascular Diseases (CVDs)

Depression and mental health disorders have a profound impact on CVDs, influencing both the development and prognosis of these conditions. Depression is a well-established risk factor for CVDs, with numerous studies highlighting its role in increasing the incidence of coronary artery disease, hypertension, and arrhythmias [14]. The bidirectional relationship between depression and CVDs creates a complex clinical challenge, as each condition can exacerbate the other.

Physiological mechanisms linking depression to CVDs include dysregulation of the autonomic nervous system, increased inflammatory markers, endothelial dysfunction, and hyperactivation of the hypothalamic-pituitary-adrenal (HPA) axis [15]. These biological changes contribute to arterial stiffness, atherosclerosis, and arrhythmic events, thereby elevating cardiovascular risk.

Patients with CVDs often exhibit higher rates of depression compared to the general population. Depression in these patients is associated with poor adherence to medical regimens, unhealthy lifestyle behaviors (such as smoking, physical inactivity, and poor diet), and reduced engagement in rehabilitation programs [16]. These factors collectively impair disease management and lead to worse clinical outcomes, including increased morbidity and mortality.

The psychological burden of living with a chronic cardiovascular condition, coupled with the fear of sudden complications, significantly affects patients’ mental health. This creates a vicious cycle where mental distress worsens cardiovascular health, and deteriorating cardiovascular health further impacts mental well-being [17].

Assessing and addressing depression in patients with CVDs is essential for comprehensive care. Tools such as the PHQ-9 allow clinicians to identify and monitor depressive symptoms, enabling timely interventions [18]. Integrating mental health support into cardiovascular care plans can improve treatment adherence, enhance QoL, and reduce the overall burden of CVDs.

### 1.3. Importance of SF-36 and PHQ-9 Questionnaires in Assessing QoL

The SF-36 and PHQ-9 questionnaires are essential tools in evaluating the QoL and mental health of patients with chronic conditions, including cardiac arrhythmias, due to their comprehensive and validated assessment mechanisms.

The SF-36, developed through the Medical Outcomes Study at the New England Medical Center, is designed to measure QoL across eight critical dimensions: physical functioning, bodily pain, general health perceptions, vitality, social functioning, emotional well-being, and role limitations due to physical and emotional health issues [19]. Its structure includes 36 items aggregated into two summary measures: the physical component summary (PCS) and the mental component summary (MCS) [20]. Each dimension is scored from 0 to 100, with higher scores indicating better health status. The SF-36 has demonstrated high reliability and validity across various populations and diseases, including its validated Romanian version, ensuring accurate assessments in diverse clinical settings. Its wide application in clinical trials and research studies underscores its ability to capture subtle changes in patients’ perceived health over time.

PHQ-9 serves as a concise and effective screening tool for depression, aligned with DSM-IV criteria for major depressive disorder. Its nine items cover symptoms such as anhedonia, depressed mood, sleep disturbances, fatigue, appetite changes, feelings of worthlessness, concentration difficulties, psychomotor agitation/retardation, and suicidal ideation [12]. Each item is scored from 0 to 3, with total scores categorized as minimal (0–4), mild (5–9), moderate (10–14), moderately severe (15–19), and severe depression (20–27). The PHQ-9′s simplicity and rapid administration make it invaluable in both primary care and specialized settings, providing a quantifiable measure of depressive symptoms that can be tracked over time and correlated with disease progression or therapeutic outcomes [21].

The integration of these two instruments in clinical research offers a multidimensional view of patient well-being. SF-36 evaluates the physical and psychosocial impact of cardiac arrhythmias, while PHQ-9 identifies coexisting depressive symptoms that may influence disease management and prognosis. Together, they enhance the understanding of how cardiac arrhythmias affect patients’ lives beyond clinical symptoms, guiding tailored interventions to improve both mental health and overall QoL.

### 1.4. Role of AI in Analyzing Medical Data Related to Cardiac Conditions

Artificial intelligence (AI) is transforming the landscape of medical data analysis, particularly in the field of cardiology. With the exponential growth of healthcare data, AI provides advanced tools for processing, analyzing, and interpreting complex data sets, leading to more accurate diagnostics, personalized treatments, and improved patient outcomes [22].

In cardiac care, AI algorithms, including machine learning (ML) and deep learning (DL) models, are employed to detect arrhythmias, predict cardiovascular risks, and assist in decision-making processes. AI-driven analysis of electrocardiograms (ECGs) allows for the identification of subtle abnormalities that may be overlooked by human observation, enhancing early diagnosis and intervention [23].

AI also plays a critical role in risk stratification by analyzing patient demographics, clinical histories, and genetic information to predict the likelihood of adverse cardiac events [24]. This enables clinicians to implement preventive measures tailored to individual patients. Additionally, AI-powered tools assist in optimizing treatment plans by evaluating the effectiveness of various therapeutic options based on historical patient data.

Another significant application is in the management of large-scale clinical studies, where AI facilitates data integration, pattern recognition, and outcome prediction. In this study, AI-assisted statistical tools were used to merge and analyze data from SF-36 and PHQ-9 questionnaires, providing a comprehensive assessment of QoL and mental health in patients with cardiac arrhythmias [25].

**Figure 1:**
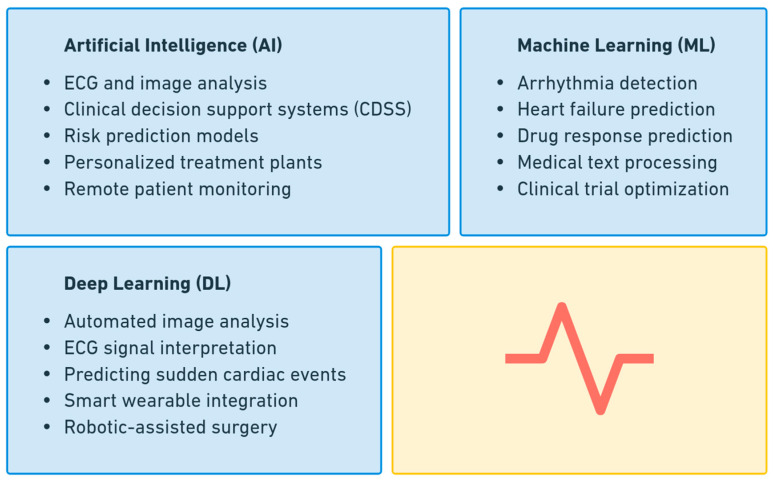
Applications of AI, ML, and DL in cardiac care.

The integration of AI in cardiology not only improves diagnostic accuracy and efficiency but also enhances patient care by enabling data-driven clinical decisions. As technology continues to evolve, AI is expected to play an increasingly vital role in advancing cardiac research, patient monitoring, and individualized treatment strategies. Figure 1 provides an overview of the key applications of AI, ML, and DL in the field of cardiac care.

## 2. Materials and Methods

### 2.1. Data Collection

This study included a total of 145 adult participants diagnosed with cardiac arrhythmias by a certified cardiologist. All participants provided informed consent to participate in the study, and ethical approval was obtained from the appropriate institutional review board. All participants were undergoing pharmacological treatment exclusively, with no individuals using pacemakers. The participants were divided into two groups:

- Group 1: Adults diagnosed with cardiac arrhythmias without any associated cardiovascular comorbidities, receiving medication-based treatment.
- Group 2: Adults diagnosed with cardiac arrhythmias accompanied by other cardiovascular conditions, also receiving medication-based treatment.

**Figure 2:**
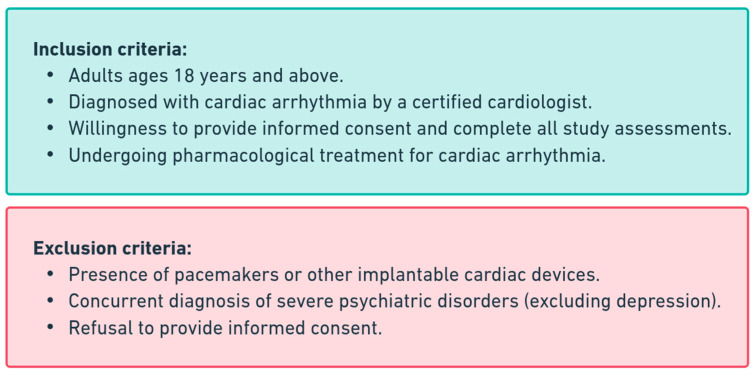
Inclusion and exclusion criteria for study participants.

Participants’ ages ranged from 24 to 80 years (mean age: 52 years), with 59 males (41%) and 86 females (59%). The study population included individuals from diverse socio-economic backgrounds, primarily from urban and suburban areas. The inclusion and exclusion criteria applied in participant selection are summarized in Figure 2.

The data set included detailed demographic data (age, gender, socioeconomic background), clinical data (type of arrhythmia, duration of the condition, presence of comorbidities such as chronic heart failure, atrial fibrillation, and hypertension), and psychological data (PHQ-9 depression scores and SF-36 QoL scores). Specific diagnoses included tachycardia, atrial fibrillation, bradycardia, and atrial ectopic beats, with durations ranging from 2 months to 12 years. Depressive symptoms were assessed using the PHQ-9 questionnaire, with 71 participants showing varying degrees of depression (mild, moderate, or severe) and 74 participants showing no signs of depression; none were on antidepressant medication. SF-36 QoL data included scores for physical functioning, role limitations due to physical health, bodily pain, general health, vitality, social functioning, role limitations due to emotional health, and emotional well-being. Data from PHQ-9 and SF-36 were collected, merged, and analyzed using AI-assisted statistical tools to ensure accuracy and robustness.

This comprehensive data set provided an in-depth understanding of the clinical, demographic, and psychological profiles of patients with cardiac arrhythmias, forming a solid foundation for subsequent analysis and interpretation.

### 2.2. Clinical Statistics

#### 2.2.1. Descriptive Statistical Analysis of Clinical Scores

Descriptive statistical methods were employed to summarize the clinical data derived from the SF-36 and PHQ-9 scores. The analysis included the calculation of means, medians, standard deviations (SD), and interquartile ranges (IQR) for both sets of scores [26]. The mean provided an overall measure of central tendency, offering insight into the average score values across the data set [26]. Complementing this, the median was used to indicate the central value, which is particularly useful in the presence of skewed data or outliers, ensuring that the representation of the central tendency is not unduly influenced by extreme values. The SD was computed to assess the dispersion of the scores around the mean, highlighting the variability within the data set [27]. A higher SD indicated greater spread in the participants’ responses, while a lower value suggested more consistency. Additionally, the IQR was calculated to measure the range within which the central 50% of the data points lie, providing a robust measure of statistical dispersion less sensitive to outliers than the overall range [28]. These descriptive statistics collectively offered a comprehensive summary of the clinical data at a basic level, setting the foundation for more advanced statistical analyses in subsequent sections.

#### 2.2.2. Normality Test: Shapiro–Wilk

To assess the normality of the clinical score distributions, the Shapiro–Wilk test was employed. This statistical test evaluates whether a given data set follows a normal distribution, a key assumption for many subsequent parametric analyses [29]. The Shapiro–Wilk test calculates a W statistic, where values close to 1 indicate that the data set is likely to be normally distributed, while values significantly lower than 1 suggest deviations from normality [30]. The corresponding *p*-value determines the statistical significance of this deviation; a *p*-value below a specified threshold (typically 0.05) indicates that the null hypothesis of normality can be rejected. The application of the Shapiro–Wilk test to the SF-36 and PHQ-9 scores provided critical insights into the underlying distribution of the clinical data. This step ensured that the choice of subsequent statistical methods was appropriately aligned with the data’s distributional characteristics, thereby enhancing the robustness and validity of the analyses conducted in later sections.

#### 2.2.3. Group Comparisons: Mann–Whitney U Test

For comparing clinical scores between patients with depression and those without, the Mann–Whitney U test was employed due to the non-normal distribution of the data, as determined by the Shapiro–Wilk test [31]. This non-parametric test is suitable for comparing two independent groups without assuming a specific data distribution, making it ideal for the clinical scores in this study. The Mann–Whitney U test ranked all observations from both groups together and evaluated whether the ranks for one group were significantly different from the ranks of the other group [32]. The test yielded a U statistic and an associated *p*-value, with a significance threshold of 0.05. A *p*-value below this threshold indicated a statistically significant difference in the distributions of clinical scores between the depression and non-depression groups. By using the Mann–Whitney U test, the analysis ensured that the comparisons were both statistically sound and appropriately suited to the data characteristics, providing reliable insights into the differences in health-related QoL across clinical groups.

#### 2.2.4. Association Analysis: Chi-Square Test

To examine the association between depression and the type of arrhythmia, the Chi-square test of independence was utilized. This non-parametric test is designed to assess whether there is a significant association between two categorical variables [33]. In this case, the categorical variables were the presence or absence of depression, as determined by the PHQ-9 scores, and the type of arrhythmia, which included chronic heart failure, atrial extrasystoles, atrial fibrillation, and tachycardia, as classified in the clinical data set [34]. The Chi-square test compares the observed frequencies in each category to the frequencies that would be expected if the variables were independent. The test yields a Chi-square statistic and a corresponding *p*-value, with statistical significance typically set at *p* < 0.05. This method allowed for the identification of potential relationships between psychological conditions such as depression and specific cardiac arrhythmias, providing valuable insights into the interplay between mental health and cardiovascular health within the patient cohort.

#### 2.2.5. Kruskal–Wallis Test for Comparing Clinical Scores Across Multiple Groups

For comparing clinical scores across multiple groups, the Kruskal–Wallis test was employed due to the non-normal distribution of the data, as determined by the Shapiro–Wilk test [35]. This non-parametric test is an extension of the Mann–Whitney U test and is used to assess whether there are statistically significant differences in the distributions of scores across three or more independent groups. In this analysis, patients were categorized into five groups based on their PHQ-9 scores: minimal (0–4), mild (5–9), moderate (10–14), moderately severe (15–19), and severe (20–27) [12]. The Kruskal–Wallis test ranked all observations from these groups together and compared the average rank of each group. The test produced an H statistic and an associated *p*-value, with statistical significance set at *p* < 0.05. A significant result indicated that at least one group’s distribution differed from the others, though further post hoc tests would be necessary to identify which specific groups were different. This method ensured that the comparison of clinical scores across multiple patient groups was both statistically sound and appropriately suited to the data characteristics, providing reliable insights into variations in health-related QoL and clinical outcomes among different levels of depression severity [36].

#### 2.2.6. Spearman’s Correlation for the Relationship Between SF-36 and PHQ-9 Scores

To examine the relationship between the SF-36 and PHQ-9 scores, Spearman’s rank correlation was employed due to the non-normal distribution of the data [37]. This non-parametric method assesses the strength and direction of the monotonic relationship between two variables by ranking the data points and calculating the correlation based on these ranks. Spearman’s correlation coefficient (ρ) ranges from −1 to +1, where values close to +1 indicate a strong positive relationship, values close to −1 indicate a strong negative relationship, and values near 0 suggest no monotonic association [38]. This method was chosen for its robustness in handling non-normally distributed data and its ability to capture nonlinear relationships, ensuring an accurate analysis of the association between health-related QoL dimensions (SF-36 scores) and the severity of depressive symptoms (PHQ-9 scores).

#### 2.2.7. Linear Regression to Analyze the Influence of Disease Duration on QoL Scores

To analyze the influence of disease duration on QoL scores, a linear regression analysis was employed. Linear regression is a statistical method used to model the relationship between a dependent variable and one or more independent variables [39]. In this case, the QoL scores measured by the SF-36 dimensions served as the dependent variables, while the duration of the disease was the independent variable. The linear regression model estimated a regression coefficient, indicating the direction and strength of the relationship between disease duration and each QoL dimension. A positive coefficient suggested that an increase in disease duration was associated with higher QoL scores, while a negative coefficient indicated an inverse relationship. This method provided a means to quantify how changes in the duration of the disease influenced the various dimensions of health-related QoL, offering a statistical framework to assess the impact of chronic illness on patients’ well-being over time.

#### 2.2.8. Factor Analysis for Identifying Common Dimensions in SF-36 and PHQ-9

To identify common underlying dimensions between the SF-36 and PHQ-9 scores, factor analysis was employed. Factor analysis is a multivariate statistical method used to uncover latent variables, or factors, that explain the observed correlations among a set of measured variables [40]. In this study, it was used to determine whether the items from the SF-36 QoL dimensions and the PHQ-9 depression scores loaded onto common factors, suggesting shared underlying constructs. The method involved calculating a correlation matrix of all SF-36 and PHQ-9 items, followed by extracting two factors, as specified in the analysis. Factor loadings were then assessed to determine which variables contributed most to each factor, and varimax rotation was applied to enhance interpretability by maximizing the variance of loadings within each factor. This analysis provided insights into how dimensions of health-related QoL and depression symptoms overlap, revealing potential shared constructs such as emotional well-being, physical functioning, and mental health status, which could aid in better understanding the interplay between physical and mental health in clinical populations.

### 2.3. Demographic Statistics

#### 2.3.1. Descriptive Analysis of Demographic Data (Age, Gender, Disease Duration)

Descriptive statistical methods were employed to summarize the demographic characteristics of the patient cohort, focusing on age, gender, and disease duration. The analysis included the calculation of measures of central tendency, such as the mean and median, to provide an overall summary of patients’ ages and disease durations [27]. Measures of dispersion, such as the SD and IQR, were used to capture the variability within these demographic variables. For categorical data, such as gender, the analysis included the computation of frequencies and percentages to illustrate the distribution of male and female participants within the cohort. This descriptive analysis provided an essential overview of the demographic composition of the study population, offering key insights into the age distribution, gender balance, and duration of illness among patients, thereby laying the groundwork for more advanced statistical analyses in subsequent sections.

#### 2.3.2. Chi-Square Test for the Relationship Between Gender/Age Groups and Depression Scores

To examine the relationship between demographic variables such as gender and age groups and depression scores (PHQ-9), the Chi-square test of independence was employed [33]. This non-parametric test is used to determine whether there is a significant association between two categorical variables. The PHQ-9 depression scores were categorized using the same severity levels as in the association analysis with arrhythmias, including minimal, mild, moderate, moderately severe, and severe. Similarly, the age variable was grouped into three categories: 20–40 years, 41–60 years, and 61–80 years to facilitate comparison. The Chi-square test compared the observed frequencies of depression severity levels across different gender and age categories to the frequencies expected if the variables were independent [34]. The test yielded a Chi-square statistic and an associated *p*-value, with a significance level typically set at *p* < 0.05. This method allowed for the exploration of potential associations between demographic factors and depression severity, providing insight into how age and gender may relate to mental health outcomes within the patient cohort.

#### 2.3.3. Trend Test for Variation of Scores Based on Age or Disease Duration

To assess the variation of clinical scores based on age and disease duration, a Kruskal–Wallis trend test was employed due to the non-normal distribution of the data [35]. This non-parametric method is used to evaluate whether there is a significant trend or ordered relationship between an ordinal variable and a continuous outcome. In this analysis, PHQ-9 depression scores were used as the continuous outcome variable, while age groups were categorized using the same intervals as in the Chi-square test for demographic data, specifically, 20–40 years, 41–60 years, and 61–80 years. The disease duration was divided into four equal intervals to facilitate the analysis. The Kruskal–Wallis test ranked the values within each group and assessed whether there was a systematic variation in PHQ-9 scores across these ordered categories [36]. By applying this method, it was possible to determine whether older age groups or longer durations of illness were associated with specific trends in depression severity, providing a nuanced understanding of how demographic factors influence clinical outcomes.

#### 2.3.4. Linear Regression to Evaluate the Impact of Demographic Factors on Clinical Scores

To evaluate the impact of demographic factors on clinical scores, a linear regression analysis was employed. This method models the relationship between one or more independent variables (predictors) and a continuous dependent variable (outcome) [39]. In this analysis, demographic factors such as age, gender, and disease duration were used as independent variables, while clinical scores from the SF-36 dimensions and PHQ-9 served as the dependent variables. The linear regression model estimated coefficients for each demographic factor, indicating the strength and direction of their influence on the clinical scores. A positive coefficient suggested that an increase in the demographic variable (e.g., older age or longer disease duration) was associated with higher clinical scores, while a negative coefficient indicated an inverse relationship. This analysis provided insights into how demographic characteristics influence health-related quality of QoL and depression severity, highlighting key predictors of clinical outcomes within the patient cohort.

#### 2.3.5. Principal Component Analysis (PCA) for Dimensionality Reduction and Identification of Relevant Demographic Variables

To reduce the dimensionality of the demographic data set and identify the most relevant variables, was employed. PCA is an unsupervised ML technique used to transform a data set with potentially correlated variables into a set of uncorrelated components, known as principal component (PC) [41]. Each PC is a linear combination of the original variables, capturing the maximum variance in the data set. In this analysis, demographic variables such as age, gender, and disease duration were standardized to ensure that all variables contributed equally to the PCs. PCA was then applied to reduce the data set into a smaller set of components while retaining as much of the original variance as possible. The PCs were ranked based on the amount of variance they explained, with the first few components capturing most of the data set’s variability. This method enabled the identification of the most relevant demographic variables by examining the component loadings, which indicate the contribution of each original variable to the PCs. PCA provided a structured approach to dimensionality reduction, facilitating further statistical analyses by focusing on the most influential demographic factors within the patient cohort [42].

### 2.4. AI-Assisted Statistics

#### 2.4.1. Cluster Analysis (k-Means) for Grouping Patients Based on Demographic Variables

To group patients based on demographic variables, k-means cluster analysis was employed [42]. K-Means is an unsupervised ML algorithm used to partition a data set into a predefined number of clusters, with each data point belonging to the cluster with the nearest mean. In this analysis, demographic variables such as age, gender, and disease duration were used as input features for clustering [43]. The algorithm iteratively assigned patients to clusters by minimizing the within-cluster variance, ensuring that patients within the same cluster were more like each other than to those in other clusters. The number of clusters was selected based on the data structure and prior considerations, and the centroids were updated iteratively until convergence was reached. This method provided a data-driven approach to identifying distinct subgroups within the patient cohort based on demographic characteristics, offering a valuable perspective on patient segmentation for further clinical analysis [44].

#### 2.4.2. Multivariable Logistic Regression for Predicting Depression Based on Clinical and Demographic Data

To predict depression based on clinical and demographic data, a multivariable logistic regression model was employed. Logistic regression is a supervised ML method used to model the probability of a binary outcome based on one or more predictor variables [45]. In this analysis, the presence or absence of depression (PHQ-9 scores ≥ 10) served as the binary dependent variable, while clinical variables from the SF-36 dimensions and demographic factors such as age, gender, and disease duration were used as independent variables. The data set was preprocessed by encoding categorical variables (e.g., gender) using Label Encoder (scikit-learn 1.6.1) and standardizing all features using Standard Scaler (scikit-learn 1.6.1). To address class imbalance, the Synthetic Minority Over-sampling Technique (SMOTE) 0.13.0 was applied only to the training set, ensuring that the model was trained on a balanced data set. Recursive Feature Elimination (RFE) (scikit-learn 1.6.1) was employed to select the top five most relevant features before training the Logistic Regression (scikit-learn 1.6.1) model. The selected features were used to train the model, and its performance was evaluated on the test set using classification metrics such as accuracy, precision, recall, and F1-score, as well as a confusion matrix. This method provided a robust approach to assessing the combined influence of clinical and demographic factors on depression risk, enhanced by SMOTE for class balance and RFE for feature selection, offering valuable insights for predictive modeling in clinical settings [46].

#### 2.4.3. XGBoost for Robust Predictive Models and Receiver-Operating Characteristic Curve (ROC) Analysis for Evaluating Performance

To develop robust predictive models for clinical data analysis, the XGBoost (2.1.4) algorithm was employed. XGBoost is an ensemble ML technique based on gradient boosting, known for its high performance, speed, and scalability [47]. In this analysis, clinical variables from the SF-36 dimensions and demographic factors such as age, gender, and disease duration were used as predictors, while the PHQ-9 depression scores served as the continuous target variable for regression. The data set was preprocessed by encoding categorical variables using Label Encoder (scikit-learn 1.6.1) and standardizing numerical features with Standard Scaler (scikit-learn 1.6.1). To optimize model performance, GridSearchCV (scikit-learn 1.6.1) was employed for hyperparameter tuning, refining parameters such as the number of estimators, maximum depth, learning rate, L1, and L2 regularization. To further evaluate the predictive performance of XGBoost, ROC analysis (scikit-learn 1.6.1) was conducted. Since the model was trained as a regression model, a threshold-based classification approach was applied, converting predicted PHQ-9 scores into binary categories using a threshold of 10 to differentiate between depressed and non-depressed individuals. The ROC curve and area under the curve (AUC) score provided additional insights into the model’s ability to distinguish between clinical depression and non-depression cases [48].

## 3. Results

### 3.1. SF-36 and PHQ-9 Scores

#### 3.1.1. SF-36

The SF-36 questionnaire provided insights into the QoL for patients with cardiac arrhythmias. Group 1 (patients with cardiac arrhythmias only) exhibited a mean physical health score of 67.72 and a mental health score of 73.79, indicating a relatively higher QoL compared to Group 2. Patients in Group 2 (those with additional cardiovascular conditions) showed lower mean scores of 61.48 for physical health and 66.37 for mental health. The SD values highlight greater variability within Group 1, possibly due to varying severities of arrhythmias and psychological states.

#### 3.1.2. PHQ-9

Depression severity, assessed through the PHQ-9, varied between the groups. Group 1 had a mean PHQ-9 score of 4.81, suggesting mild depressive symptoms on average, while Group 2 had a higher mean score of 6.19, indicating a more pronounced level of depression. The SD further reflects a wider range of depressive symptoms within Group 2, potentially due to the compounded effect of additional cardiovascular issues.

#### 3.1.3. Correlation Between Tests

Correlation analysis revealed a significant inverse relationship between depression severity and QoL, particularly in Group 1. The negative correlation between PHQ-9 and SF-36 physical health scores (−0.32) and mental health scores (−0.44) indicates that higher depression levels are associated with poorer QoL. In Group 2, the correlation between PHQ-9 and physical health was weaker (−0.25) and nearly absent with mental health (−0.04), suggesting that other health factors may overshadow the impact of depression in this group. These findings underscore the importance of addressing mental health alongside physical health in patients with cardiac arrhythmias, especially those with additional cardiovascular conditions. A summary of the SF-36 and PHQ-9 scores, along with correlation analysis, is provided in Table 1.

### 3.2. Clinical Results

#### 3.2.1. Descriptive Statistical Analysis of Clinical Scores

The mean physical functioning score was 76.77 (SD = 28.74), with a median of 90.0 and an IQR of 35.0. The mean PHQ-9 score was 5.03 (SD = 4.55), with a median of 4.0 and an IQR of 5.0. Other SF-36 dimensions showed median values of 100.0 for role limitations due to physical and emotional health, 70.0 for general health and energy, and 76.0 for emotional well-being. The IQR values ranged from 0.0 to 37.5 across different dimensions, indicating varying degrees of variability in clinical scores.

#### 3.2.2. Normality Test, Group Comparisons, and Association Analysis

The Shapiro–Wilk test demonstrated that all clinical scores had W-statistics significantly below 1 and *p*-values ≤ 0.05, indicating that none of the distributions met the criteria for normality. This deviation from normality was consistent across all measured dimensions, including physical functioning, pain, general health, energy levels, social functioning, emotional well-being, and health status change. These findings justified the use of non-parametric statistical methods in subsequent analyses to ensure the validity and reliability of the results.

The Mann–Whitney U test revealed significant differences (*p* ≤ 0.05) between patients with depression and those without across all clinical score dimensions, including physical functioning, pain, general health, energy levels, social functioning, and emotional well-being. The only dimension that did not show a statistically significant difference was health status change. These results highlight the considerable impact of depression on various aspects of health-related QoL in patients with cardiac arrhythmias.

The Chi-square test indicated no significant association (*p* > 0.05) between depression and any type of arrhythmia, including chronic heart failure, atrial extrasystoles, atrial fibrillation, and tachycardia.

#### 3.2.3. Kruskal–Wallis Test, and Spearman’s Correlation

The Kruskal–Wallis test revealed significant differences (*p* ≤ 0.05) in all SF-36 dimensions across the five PHQ-9 depression severity categories. The clinical dimensions that showed statistically significant variation included physical functioning, role limitations due to physical and emotional health, pain, general health, energy/fatigue, social functioning, emotional well-being, and perceived health change. These results suggest that the severity of depression is associated with variations in multiple aspects of health-related QoL, highlighting the impact of psychological health on clinical outcomes in patients with cardiac arrhythmias.

Spearman’s correlation analysis revealed significant negative correlations (*p* ≤ 0.05) between PHQ-9 scores and all SF-36 dimensions. The strongest negative correlations were observed in energy/fatigue (ρ = −0.45), social functioning (ρ = −0.47), and role limitations due to emotional problems (ρ = −0.48). These results indicate that higher levels of depressive symptoms are associated with lower health-related QoL across physical, emotional, and social dimensions, highlighting the substantial impact of depression on patients with cardiac arrhythmias.

#### 3.2.4. Linear Regression to Analyze the Influence of Disease Duration on QoL Scores

Descriptive analysis showed that the mean age of patients was 51.27 years (SD = 12.11), with an IQR of 16.5 years. The mean disease duration was 3.04 years (SD = 2.70), with an IQR of 3.0 years. The sample consisted of 102 females and 41 males. These demographic and clinical characteristics provided the basis for the linear regression analysis, which aimed to evaluate the influence of disease duration on QoL scores. A summary of clinical results, including statistical analyses, is presented in Table 2.

### 3.3. Demographic Characteristics

#### 3.3.1. Descriptive Analysis of Demographic Data (Age, Gender, Disease Duration)

The descriptive analysis revealed that the patient cohort had a mean age of 51.27 years (SD = 12.11), with ages ranging from 24 to 80 years. The IQR of 16.5 years indicates a moderately wide age distribution, with 50% of the patients aged between 43 and 59.5 years. The mean duration of disease was 3.04 years (SD = 2.70), with a range from 1 to 12 years, and an IQR of 3.0 years, highlighting variability in the duration of illness among the patients. The gender distribution showed that the majority of the cohort were female (102 patients, 71.3%), while males accounted for 41 patients (28.7%). This demographic overview provides a clear understanding of the patient population’s composition and serves as a foundation for further statistical analyses in subsequent sections.

#### 3.3.2. Chi-Square Test for the Relationship Between Gender/Age Groups and Depression Scores

The Chi-square test for independence revealed no significant association between gender and depression severity levels (χ^2^= 2.36, *p* = 0.67), indicating that depression scores were independent of gender within the patient cohort. However, a significant association was found between age groups and depression severity levels (χ^2^ = 16.30, *p* = 0.038). This suggests that age may play a role in the severity of depressive symptoms, with notable variations in depression scores observed across the three age categories (20–40 years, 41–60 years, and 61–80 years). The results highlight that older patients may experience different levels of depression compared to younger groups, emphasizing the importance of considering age in the psychological assessment and management of cardiac arrhythmia patients.

#### 3.3.3. Trend Test for Variation of Scores Based on Age or Disease Duration

The Kruskal–Wallis trend test revealed a significant variation in PHQ-9 depression scores across age groups (χ^2^ = 8.46, *p* = 0.015), indicating that depression severity varied systematically with age. This suggests a potential trend where older age groups may experience different levels of depressive symptoms compared to younger patients. However, no significant trend was observed in PHQ-9 scores based on disease duration (χ^2^ = 7.11, *p* = 0.068), suggesting that the duration of illness did not have a statistically significant influence on the severity of depression in this patient cohort.

#### 3.3.4. Linear Regression to Evaluate the Impact of Demographic Factors on Clinical Scores

The linear regression analysis demonstrated that age, gender, and disease duration had varying impacts on clinical scores. Age was identified as a significant predictor of PHQ-9 depression scores (χ^2^ = 8.46, *p* = 0.015), indicating that older age was associated with higher depression severity. However, disease duration did not show a statistically significant impact on PHQ-9 scores (χ^2^ = 7.11, *p* = 0.068). These findings highlight that age plays a critical role in influencing depression severity among patients with cardiac arrhythmias, while disease duration may not have a direct impact on clinical outcomes related to mental health.

#### 3.3.5. PCA for Dimensionality Reduction and Identification of Relevant Demographic Variables

PCA revealed that the first three PCs explained 58.65%, 26.92%, and 14.43% of the total variance, respectively, capturing almost the entire variability in the demographic dataset. Among the demographic variables, disease duration, and gender were identified as the most relevant contributors to the PCs, indicating that these factors played a significant role in the demographic structure of the patient cohort. PCA effectively reduced the dimensionality while preserving critical information, highlighting disease duration and gender as key demographic factors influencing clinical outcomes.

A summary of the demographic characteristics and statistical analyses is provided in Table 3.

### 3.4. AI Analysis: Models, Performance, Validation

#### 3.4.1. K-Means for Grouping Patients Based on Demographic Variables

**Figure 3:**
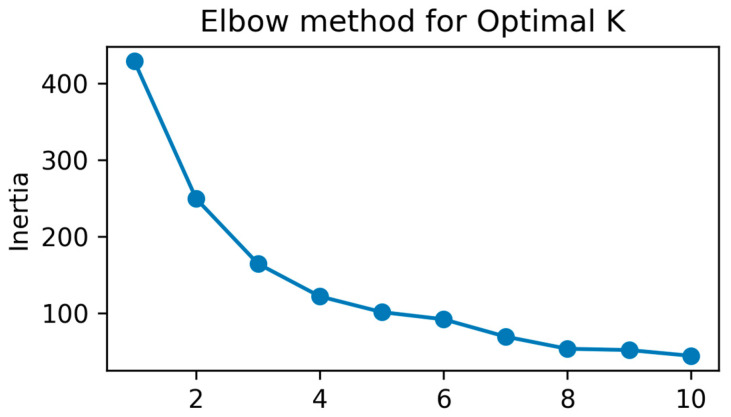
Elbow method for determining the optimal number of clusters (K) in k-means clustering.

The k-means clustering algorithm grouped the patient cohort into three distinct clusters based on age, gender, and disease duration. The optimal number of clusters (k = 3) was determined using the elbow method, as shown in Figure 3. The plot displays a clear “elbow” at k = 3, where the inertia (within-cluster variance) starts to level off, indicating that adding more clusters would result in minimal improvement.

The clusters were distributed as follows:

- Cluster 0: 34 patients with an average age of 47.7 years, predominantly female (gender mean close to 0), and a mean disease duration of 1.91 years.
- Cluster 1: 93 patients with an average age of 52.9 years, predominantly male (gender mean = 1), and a mean disease duration of 3.24 years.
- Cluster 2: 16 patients with an average age of 68.4 years, a mixed gender distribution (mean = 0.44), and a notably longer mean disease duration of 9.19 years.

These clusters highlight the presence of distinct patient sub-groups within the cohort, providing valuable insights for targeted clinical interventions. The differences in age, gender, and disease duration across the clusters suggest potential variations in clinical needs, treatment responses, and health outcomes.

#### 3.4.2. Multivariable Logistic Regression for Predicting Depression Based on Clinical and Demographic Data

The logistic regression model demonstrated strong predictive performance, achieving an overall accuracy of 93%, as shown in Table 4. The confusion matrix revealed that the model correctly identified most cases, as reflected in the performance metrics table.

**Figure 4:**
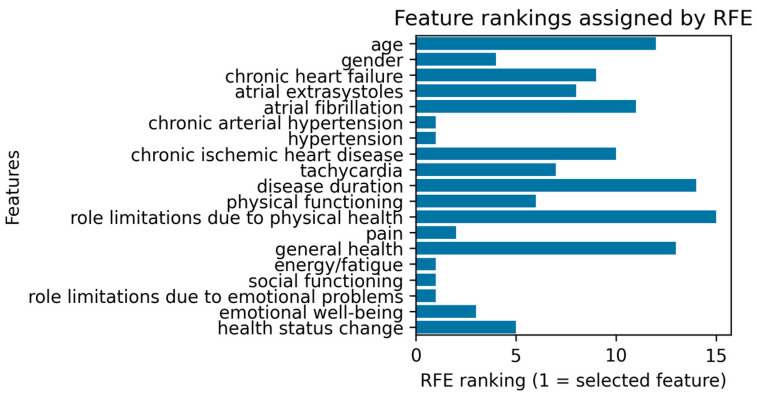
Key predictors for depression classification identified by RFE.

Precision values of 1.00 for the non-depressed class and 0.60 for the depressed class indicated that all positive predictions for the non-depressed group were correct, while there were some false positives for the depressed group. Recall values of 0.92 and 1.00, respectively, highlighted the model’s excellent ability to minimize false negatives, particularly for depression detection. The F1-scores of 0.96 for the non-depressed class and 0.75 for the depressed class further confirmed the model’s reliability, providing an overall balanced performance in classifying patients based on their clinical and demographic profiles. Additionally, the implementation of RFE, as shown in Figure 4, identified the most relevant predictors for depression classification: chronic arterial hypertension, HTA, fatigue/energy levels, social functioning, and role limitations due to emotional problems.

#### 3.4.3. XGBoost for Robust Predictive Models and ROC Analysis for Evaluating Performance

**Figure 5:**
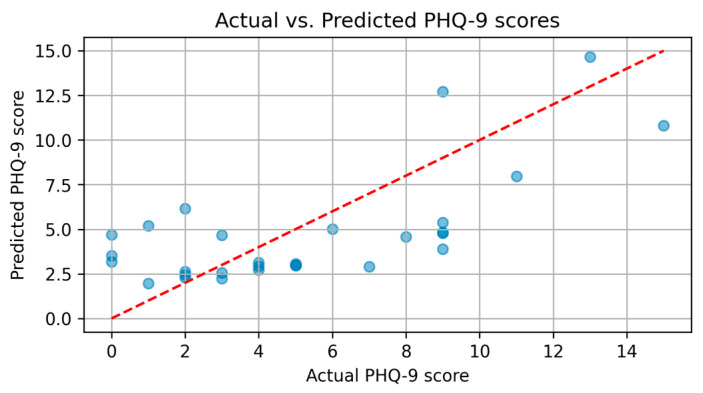
Scatter plot of actual vs. predicted PHQ-9 scores using XGBoost.

The XGBoost model demonstrated strong predictive capability, effectively capturing patterns within the clinical and demographic data. The scatter plot of actual versus predicted PHQ-9 scores, as shown in Figure 5, highlights the model’s ability to estimate depression severity with reasonable accuracy. While most predictions align closely with actual values, some deviations, particularly for extreme cases, suggest that additional refinements could further improve performance.

**Figure 6:**
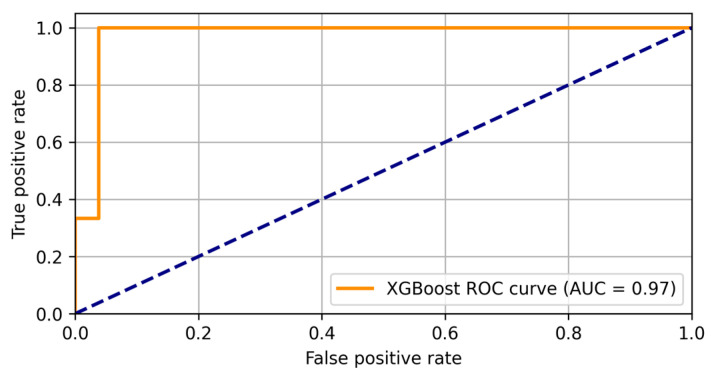
ROC curve for XGBoost PHQ-9 regression model.

Each blue dot in the figure represents an individual case, where the x-coordinate indicates the actual PHQ-9 score and the y-coordinate shows the corresponding score predicted by the XGBoost model. The proximity of the dots to the red dashed diagonal line (representing perfect predictions) reflects the accuracy of the model — points closer to the line indicate more accurate predictions, whereas points further away suggest higher prediction errors. These discrepancies are likely due to the limited data set, which may not fully encompass the variability of higher PHQ-9 scores. Expanding the data set could enhance the model’s generalization, ensuring more reliable predictions across all severity levels. The ROC analysis, as presented in Figure 6, confirms the discriminative power of the XGBoost model in distinguishing between patients with and without depression, achieving an AUC of 0.97.

The high AUC score reflects the model’s strong classification ability, meaning it effectively differentiates between the two groups. The curve indicates that the model achieves a balance between sensitivity and specificity, minimizing false positives and false negatives. The dotted blue diagonal line represents the performance of a random classifier, which would have no discriminative power. The further the ROC curve lies above this diagonal, the better the model’s performance. In this case, the XGBoost model’s curve lies well above the diagonal, indicating excellent predictive accuracy. Overall, the results highlight XGBoost as a powerful tool for clinical predictive modeling, capable of providing meaningful insights into depression severity. With further data expansion and refinement, it has the potential to become an even more robust and reliable method for supporting clinical assessments and decision-making.

Table 5 summarizes the key findings and performance metrics of the AI models used in this study.

### 3.5. Additional Age-Stratified Analysis

An additional analysis was performed to investigate how age modifies the relationship between cardiac arrhythmias, depression (PHQ-9), and overall QoL (SF-36). The total sample (*n =* 145) was divided into three age groups: 20–40 years (*n =* 40), 41–60 years (*n =* 70), and over 60 years (*n =* 35). Statistical comparisons among age categories were conducted using the Kruskal–Wallis test for continuous variables (PHQ-9 scores, SF-36 subscales) and Mann–Whitney U tests for pairwise post hoc comparisons where indicated. Spearman’s rank correlation was calculated between age, disease duration, and PHQ-9/SF-36 dimensions to assess monotonic relationships. Logistic regression models were also explored to determine if older age remained a significant predictor of depression risk, adjusting for comorbidities, disease duration, and gender where sample sizes allowed.

#### 3.5.1. Multivariable Logistic Regression for Predicting Depression Based on Clinical and Demographic Data

- Ages 20–40 (*n =* 40): The mean PHQ-9 score in this younger subgroup was 3.8 (SD = 2.2), reflecting generally mild depressive symptoms. Correspondingly, SF-36 domain scores were relatively high, with physical health averaging around *73.4* and mental health around 76.1. Vitality and emotional well-being also scored notably better than in older age categories.
- Ages 41–60 (*n =* 70): Participants in this middle-aged range showed a moderate rise in PHQ-9 scores (mean 5.2, SD = 3.6) compared to the youngest group, coupled with slightly lower SF-36 scores—particularly in physical functioning (about 67.9) and energy/fatigue. Although these differences were not profoundly large, they suggest a progressive impact of age on QoL and depressive symptoms. AI-powered analytics: The incorporation of machine learning enhanced predictive accuracy and patient subgroup identification.
- Ages > 60 (*n =* 35): Older adults in this category had the highest average PHQ-9 score, around 7.1 (SD = 4.0), indicating mild-to-moderate depressive symptomatology, as well as the lowest SF-36 domain scores (physical health often near 60.3 and mental health around 62.9). Statistically significant differences (*p* < 0.05) emerged compared with the 20–40 age group across multiple SF-36 dimensions, underscoring a greater vulnerability to both depression and reduced QoL in later life.

These trends are consistent with broader research indicating an elevated psychological burden and poorer perceived health status in older populations with chronic conditions. However, the sample sizes within certain age brackets (especially over 60) impose limitations on making definitive statements about the strength of these age-related effects across all arrhythmia subtypes.

#### 3.5.2. Implications and Future Directions

The age-stratified results suggest that older adults with cardiac arrhythmias may require more integrated care strategies, including focused psychological interventions and comprehensive management of comorbidities. Longitudinal data collection could help clarify whether depressive symptoms and QoL declines progress more rapidly at older ages. Additionally, larger and more evenly distributed samples are recommended to enhance statistical power and ensure broader generalizability. Future work may also integrate wearable device monitoring and advanced artificial intelligence methods to capture subtler age-related changes in cardiac health and mental well-being.

## 4. Discussion

The findings of this study provide valuable insights into the impact of QoL on cardiac arrhythmias. Our results emphasize how QoL, as measured through SF-36, and psychological distress, as assessed by PHQ-9, influence both clinical outcomes and patient well-being. The interplay between cardiac arrhythmias, depression, and overall health underscores the need for an integrated, multidimensional approach to treatment.

### 4.1. The Impact of QoL on Cardiac Arrhythmias

QoL is a critical determinant of health outcomes in patients with cardiac arrhythmias. Our findings revealed that patients with additional cardiovascular comorbidities exhibited significantly lower SF-36 scores, indicating poorer physical and mental well-being. The presence of depressive symptoms further exacerbated this decline in QoL, with higher PHQ-9 scores correlating with worse SF-36 outcomes. This suggests that beyond the physiological burden of arrhythmias, emotional and psychological distress play a substantial role in disease progression and patient experience [49].

The study highlights the bidirectional relationship between QoL and arrhythmia severity. Reduced QoL, characterized by fatigue, functional limitations, and psychological distress, may contribute to increased arrhythmia symptom burden. Conversely, recurrent arrhythmic episodes can further deteriorate QoL by inducing anxiety, reducing physical activity, and diminishing social participation. These findings reinforce the importance of addressing both physical and mental aspects of health in arrhythmia management.

### 4.2. AI-Driven Insights into QoL and Cardiac Arrhythmias

AI-based analytical methods provided deeper insights into how QoL affects cardiac arrhythmia outcomes. Machine learning models successfully identified distinct patient subgroups with varying levels of QoL impairment, depression, and clinical severity. The K-means clustering algorithm segmented patients based on demographic and disease characteristics, revealing that older individuals and those with prolonged disease duration reported significantly lower QoL scores.

The logistic regression model achieved 93% accuracy in predicting depression in arrhythmia patients, highlighting the predictive power of SF-36 and clinical parameters in mental health assessment. Additionally, XGBoost classification demonstrated a strong discriminatory ability (AUC = 0.97) in detecting at-risk patients, further reinforcing the relevance of AI-assisted methodologies in clinical decision-making.

### 4.3. Clinical Implications

The integration of QoL assessments into routine cardiac care is essential for optimizing patient management. Our study findings support the implementation of routine SF-36 and PHQ-9 evaluations in arrhythmia clinics, as these tools provide critical insights into patient well-being beyond standard cardiovascular assessments. Addressing mental health through early screening and targeted interventions can significantly improve long-term outcomes.

Personalized treatment approaches should be tailored based on AI-derived patient segmentation. For instance:

- Patients with mild arrhythmias but high depression scores (Cluster 0) may benefit from cognitive-behavioral therapy and psychosocial support;
- Patients with multiple cardiovascular comorbidities and low SF-36 scores (Cluster 2) may require comprehensive lifestyle interventions alongside pharmacological treatment;
- Middle-aged patients with moderate arrhythmia burden (Cluster 1) may need a combination of lifestyle modifications and pharmacological therapy.

### 4.4. AI Integration and Future Perspectives in Daily Practice

A key strength and novelty of this study lies in the AI-driven approach used to detect and predict depression and poor QoL in patients with cardiac arrhythmias. Traditional research has long documented the connection between arrhythmias—especially atrial fibrillation—and elevated depression or diminished QoL [50,51,52]. However, few studies have incorporated robust ML and AI-based models to prospectively identify high-risk individuals and propose tailored interventions in real time.

#### 4.4.1. Utility of AI for Early Detection and Management

Our findings underscore how XGBoost and logistic regression can enhance clinical workflows by rapidly identifying patients with suboptimal mental health profiles, which might otherwise remain undetected. This predictive capability empowers clinicians to:

- Stratify risk: Instead of relying solely on periodic clinical evaluation, AI models provide continuous updates on which individuals are at higher risk for depression or deteriorating QoL.
- Guide personalized care: By highlighting variables most influential to depression (e.g., fatigue, role limitations due to emotional problems), the models can inform targeted interventions such as cognitive behavioral therapy, counseling, or closer monitoring for arrhythmia decompensations.
- Optimize resource allocation: Healthcare systems can concentrate psychosocial support on patients with the greatest immediate need, thereby improving outcomes and potentially reducing hospital admissions.

Moreover, telehealth platforms could integrate these predictive models, offering real-time flags for patients who deviate from expected QoL trajectories based on continuous data from wearable devices (e.g., heart rate, physical activity). Such an approach aligns with emerging trends in remote monitoring, facilitating timely alerts when depressive symptoms worsen or arrhythmia risk increases.

#### 4.4.2. Incorporating AI into Routine Practice

To translate these findings into daily clinical practice, the following steps may be considered:

- Implementation in EHR systems: Embedding logistic regression and XGBoost algorithms directly into EHRs to automatically evaluate PHQ-9 or SF-36 data once entered.
- Interdisciplinary collaboration: Encouraging psychiatrists, psychologists, and cardiologists to jointly review AI-generated risk scores, ensuring comprehensive care plans.
- Patient education and engagement: Providing user-friendly dashboards or mobile apps that visualize risk levels and recommended actions, motivating patients to adhere to therapy and lifestyle modifications.
- Continuous refinement: Periodic re-training of models with new patient data to maintain predictive accuracy and adapt to evolving patient profiles over time.

Such an approach aligns with emerging trends in remote monitoring, facilitating timely alerts when depressive symptoms worsen or arrhythmia risk increases.

#### 4.4.3. Limitations and Outlook

While the current study demonstrates promising results, several factors limit broad generalization. First, the cross-sectional nature and modest sample size (*n =* 145) constrain our ability to make definitive statements about long-term outcomes. A larger cohort with longitudinal follow-up is essential to confirm whether AI predictions consistently align with actual progression of depression and arrhythmia severity over time. Second, the reliance on self-reported PHQ-9 and SF-36 scales may introduce subjectivity and recall bias, although these instruments are widely validated in clinical research. Third, we did not integrate continuous physiological data (e.g., ECG-based wearable trackers); incorporating such real-time inputs could further enhance model accuracy and early warning capabilities.

Future studies should expand the sample to diverse populations with varied arrhythmia subtypes, incorporate longitudinal data collection, and explore more advanced AI techniques, such as DL architectures. By merging psychosocial assessments with continuous ECG or wearable-based metrics, researchers could develop dynamic models that not only predict risk but also autonomously recommend clinical interventions or telehealth consultations when thresholds are exceeded.

In summary, while prior work has already linked arrhythmias to depression and reduced QoL, this study illustrates how AI can enhance early detection, individualized interventions, and ongoing management. The integration of ML models in everyday practice may ultimately support better mental health outcomes, mitigate arrhythmia exacerbations, and lower healthcare burdens. By addressing current limitations and continuing to refine predictive algorithms, AI holds substantial promise in transforming the care of patients with cardiac arrhythmias.

## 5. Conclusions

This study underscores the profound impact of QoL on cardiac arrhythmias, demonstrating that reduced QoL and heightened depressive symptoms contribute to worsening clinical outcomes. Integrating mental health assessments into routine arrhythmia management, supported by AI-driven analytics, offers a novel approach to personalized cardiac care.

Future research should explore the longitudinal impact of QoL on arrhythmia outcomes, incorporating continuous patient monitoring through wearable technology. Expanding AI models with multimodal data sources—including genetic markers, ECG analysis, and biometrics—could enhance prediction accuracy and risk stratification.

Additionally, interventional studies evaluating the effectiveness of integrated mental health and cardiac care strategies are warranted. Tailored interventions, such as lifestyle counseling, digital health solutions, and AI-driven treatment recommendations, may further improve patient-centered care. Future advancements in AI, wearable technology, and mental health interventions will refine our ability to optimize patient well-being and long-term cardiovascular health.

## Figures and Tables

**Table 1:** Summary of SF-36 and PHQ-9 results in patients with cardiac arrhythmias.

Subsection	Analysis/Method	Results
3.1.1. SF-36	SF-36 questionnaire analysis	Group 1: Physical health = 67.72 (SD), Mental health = 73.79 (SD);
		Group 2: Physical health = 61.48 (SD), Mental health = 66.37 (SD)
3.1.2. PHQ-9	PHQ-9 depression severity analysis	Group 1: Mean PHQ-9 score = 4.81 (mild);
		Group 2: Mean PHQ-9 score = 6.19 (moderate)
3.1.3. Correlation between tests	Correlation analysis between SF-36 and PHQ-9	Negative correlation: PHQ-9 & SF-36 Physical Health (−0.32); PHQ-9 & SF-36 Mental Health (−0.44)

**Table 2:** Summary of clinical results and statistical analyses in patients with cardiac arrhythmias.

Section	Analysis/Method	Results
3.2.1. Descriptive statistical analysis	Descriptive statistics	Physical functioning: Mean = 76.77 (SD = 28.74), Median = 90.0, IQR = 35.0; PHQ-9: Mean = 5.03 (SD = 4.55), Median = 4.0, IQR = 5.0
3.2.2. Normality test, group comparisons, and association analysis	Shapiro–Wilk test	All clinical scores non-normal (*p* ≤ 0.05)
	Mann–Whitney U test	Significant differences (*p* ≤ 0.05) between patients with and without depression across all SF-36 dimensions except health status change.
	Chi-square test	No significant association (*p* > 0.05) between depression and arrhythmia types
3.2.3. Kruskal–Wallis test, and Spearman’s correlation	Kruskal–Wallis test	Significant differences (*p* ≤ 0.05) in all SF-36 dimensions across PHQ-9 categories
	Spearman’s correlation	Significant negative correlations (*p* ≤ 0.05) between PHQ-9 and SF-36 dimensions, strongest in energy/fatigue (ρ = −0.45), social functioning (ρ = −0.47), and role limitations due to emotional problems (ρ = −0.48)
3.2.4. Linear regression analysis	Regression model	Disease duration influenced QoL scores; Mean disease duration = 3.04 years (SD = 2.70), Mean age = 51.27 years (SD = 12.11), Sample: 102 females, 41 males

**Table 3:** Summary of demographic characteristics and statistical analyses.

Section	Analysis/Method	Results
3.3.1. Descriptive analysis of demographic data	Descriptive statistics (age, gender, disease duration)	Mean age: 51.27 years (SD = 12.11), Range: 24–80 years, IQR: 16.5 years; Mean disease duration: 3.04 years (SD = 2.70), Range: 1–12 years, IQR: 3.0 years; Gender: Female (102, 71.3%), Male (41, 28.7%)
3.3.2. Chi-square test	Association between gender, age groups, and depression severity	No significant association between gender and depression (χ^2^ = 2.36, *p* = 0.67); Significant association between age groups and depression severity (χ^2^ = 16.30, *p* = 0.038)
3.3.3. Trend test	Kruskal–Wallis test for variation of PHQ-9 scores by age and disease duration	Significant variation in depression scores by age (χ^2^ = 8.46, *p* = 0.015); No significant variation based on disease duration (χ^2^ = 7.11, *p* = 0.068)
3.3.4. Linear regression analysis	Impact of demographic factors on clinical scores	Age significantly predicted PHQ-9 scores (χ^2^ = 8.46, *p* = 0.015); Disease duration had no significant effect on PHQ-9 scores (χ^2^ = 7.11, *p* = 0.068)
3.3.5. PCA	Dimensionality reduction to identify key demographic variables	First three PCs explained 58.65%, 26.92%, and 14.43% of variance; Disease duration and gender were the most influential demographic factors.

**Table 4:** Performance metrics of multivariable logistic regression model for predicting depression.

Metric	Class 0 (No Depression)	Class 1 (Depression)	Macro Average	Weighted Average
Precision	1.00	0.60	0.80	0.96
Recall	0.92	1.00	0.96	0.93
F1-score	0.96	0.75	0.85	0.94
Accuracy				0.93

**Table 5:** Summary of AI models and their performance in predicting depression and identifying patient clusters.

AI Model	Key Findings	Performance Metrics
3.4.1. K-means clustering	Identified three patient clusters based on age, gender, and disease duration.	Cluster 0: Age 47.7, Disease Duration 1.91 years, Predominantly Female
		Cluster 1: Age 52.9, Disease Duration 3.24 years, Predominantly Male
		Cluster 2: Age 68.4, Disease Duration 9.19 years, Mixed Gender
3.4.2. Logistic regression	Achieved 93% accuracy in predicting depression based on clinical and demographic data.	Precision: 1.00 (No depression), 0.60 (Depression)
		Recall: 0.92 (No depression), 1.00 (Depression)
		F1-score: 0.96 (No depression), 0.75 (Depression)
3.4.3. XGBoost regression	Achieved AUC of 0.97, demonstrating strong predictive capability for depression severity.	AUC: 0.97 Strong classification ability, effectively distinguishing between depressed and non-depressed patients.

## Data Availability

The data supporting the reported results can be obtained upon reasonable request from the corresponding authors, provided that a clear justification for their necessity in a study is presented. However, the data cannot be made publicly available due to ethical restrictions and privacy concerns related to the sensitive nature of the medical information included in the data set.

## Abbreviations

The following abbreviations are used in this manuscript:

AI	Artificial intelligence
AUC	Area under the curve
CVDs	Cardiovascular diseases
DL	Deep learning
ECG	Electrocardiogram
HPA	Hypothalamic-pituitary-adrenal
HTA	Hypertension (arterial)
IQR	Interquartile range
K-means	K-means clustering
ML	Machine learning
MCS	Mental component summary (from SF-36)
PCA	Principal component analysis
PCS	Physical component summary (from SF-36)
PHQ-9	Patient health questionnaire-9
QoL	Quality of life
ROC	Receiver operating characteristic
RFE	Recursive feature elimination
SD	Standard deviation
SF-36	Short form-36 health survey
SMOTE	Synthetic minority over-sampling technique
XGBoost	Extreme gradient boosting
